# Bringing to light the physiological and pathological firing patterns of human induced pluripotent stem cell-derived neurons using optical recordings

**DOI:** 10.3389/fncel.2022.1039957

**Published:** 2023-01-17

**Authors:** Therese C. Alich, Pascal Röderer, Balint Szalontai, Kurt Golcuk, Shahan Tariq, Michael Peitz, Oliver Brüstle, Istvan Mody

**Affiliations:** ^1^Institute of Experimental Epileptology and Cognition Research, Medical Faculty, University Hospital Bonn, Bonn, Germany; ^2^Institute of Reconstructive Neurobiology, Medical Faculty, University Hospital Bonn, Bonn, Germany; ^3^Cellomics Unit, LIFE & BRAIN GmbH, Bonn, Germany; ^4^Cell Programming Core Facility, Medical Faculty, University of Bonn, Bonn, Germany; ^5^Department of Neurology, David Geffen School of Medicine at UCLA, Los Angeles, CA, United States

**Keywords:** iPSC-derived sensory neurons, dark quencher genetically encoded voltage indicator, action potential firing patterns, inherited erythromelalgia, GABA, glutamate, co-cultures, synchronous burst firing

## Abstract

Human induced pluripotent stem cells (hiPSCs) are a promising approach to study neurological and neuropsychiatric diseases. Most methods to record the activity of these cells have major drawbacks as they are invasive or they do not allow single cell resolution. Genetically encoded voltage indicators (GEVIs) open the path to high throughput visualization of undisturbed neuronal activity. However, conventional GEVIs perturb membrane integrity through inserting multiple copies of transmembrane domains into the plasma membrane. To circumvent large add-ons to the plasma membrane, we used a minimally invasive novel hybrid dark quencher GEVI to record the physiological and pathological firing patterns of hiPSCs-derived sensory neurons from patients with inherited erythromelalgia, a chronic pain condition associated with recurrent attacks of redness and swelling in the distal extremities. We observed considerable differences in action potential firing patterns between patient and control neurons that were previously overlooked with other recording methods. Our system also performed well in hiPSC-derived forebrain neurons where it detected spontaneous synchronous bursting behavior, thus opening the path to future applications in other cell types and disease models including Parkinson’s disease, Alzheimer’s disease, epilepsy, and schizophrenia, conditions associated with disturbances of neuronal activity and synchrony.

## 1. Introduction

Induced pluripotent stem cell (iPSC)-derived neurons represent a promising tool for modeling human neuropsychiatric and neurological diseases *in vitro* (Okano and Yamanaka, 2014). The possibility to generate unlimited numbers of disease and patient-specific neurons has been exploited to study a variety of pathological phenotypes ranging from epigenetic alterations and changes in gene expression to morphological, subcellular and biochemical pathologies. While a number of studies uncovered disease-specific changes in neuronal function, high-resolution assessments of neuronal activity and connectivity have remained challenging.

Action potential (AP) firing patterns, consisting of single spikes or complex bursting activity, are essential for communication between neurons and within neuronal networks. Temporal features of spike trains are critical for the communication between pre- and postsynaptic neurons. If neurons fire in bursts, information transfer within neuronal networks is augmented due to the enhanced probability of inducing postsynaptic neurons to fire APs (Lisman, 1997; Izhikevich et al., 2003) and increases the likelihood of synaptic potentiation (Pike et al., 1999). Yet, accurate und undisturbed detection of AP firing of single identified neurons, including fast successive APs within bursts, constitutes a major challenge. Finding an ideal method suited for monitoring the activity of hiPSC-derived neurons is difficult, as it should have single cell resolution and should be suitable for high throughput analyses, in order to account for heterogeneous populations of neurons. Furthermore, neuronal activity comprises many facets from hyperpolarization, subthreshold events to high-frequency AP trains, which should all be mapped by the method of choice. Ideally, the method should be non-invasive to not distort the activity of the monitored neurons. Electrophysiological methods are still the most widely used approaches to assess hiPSC-derived neuronal activity: multi electrode arrays (Thomas et al., 1972) can display the activity of whole neuronal ensembles, but cannot adequately resolve APs of single identified neurons, failing to discriminate different cellular subtypes. Patch-clamp techniques display the whole spectrum of neuronal activity at a single cell level (Neher and Sakmann, 1976) but are inherently time consuming, and invasive, as the interior of the cell is dialyzed by the intracellular solution in the patch pipette, which may have unpredictable effects on physiological intracellular events.

Genetically encoded optical activity sensors enable the targeting of specific neuronal subtypes and make possible the concomitant visualization of cellular ensembles. Genetically encoded calcium Indicators (GECIs) are the most widely used genetically encoded optical activity sensors and have been used in hiPSC-derived neuronal models (Real et al., 2018; Samarasinghe et al., 2021). However, GECIs have major drawbacks as they are only an indirect measure of neuronal activity, do not allow monitoring the whole spectrum of neuronal activity such as hyperpolarization and subthreshold events, and are too slow to resolve high frequency APs. Importantly, GECIs also act as calcium buffers and thereby interfere with intracellular calcium signaling (McMahon and Jackson, 2018), which can alter physiological firing patterns of neurons.

In contrast to GECIs, genetically encoded voltage indicators (GEVIs) directly and rapidly measure the electrical potential across the plasma membrane and thus constitute a desirable method to monitor neuronal activity. A major challenge associated with GEVIs is the minimal space, restricted to the plasma membrane, available for sensor molecule placement allowing for three orders of magnitude less GEVI molecules per cell than GECI molecules located in the cytoplasm (Peron et al., 2015). This paucity of GEVI molecules leads to the need for high illumination intensities in turn causing rapid photobleaching and phototoxic effects (Carlton et al., 2010). The sensor molecules in most GEVIs (Bando et al., 2019a,b; Kannan et al., 2019; Milosevic et al., 2020) are protein transmembrane domains, which span the plasma membrane and change the electrical properties of the cell increasing capacitance and decreasing membrane resistance (Akemann et al., 2009; Cao et al., 2013).

Here we use a recently published dark quencher hybrid GEVI (dqGEVI) method for the undisturbed visualization of hiPSC-derived neuronal activity. Compared to other GEVIs, the hybrid dqGEVI is non-invasive and preserves membrane integrity (Alich et al., 2021). The approach circumvents the insertion of transmembrane domains into the cell membrane by using a fluorescent protein tagged onto the outer leaflet of the plasma membrane through a glycosylphosphatidylinositol (GPI)-anchor. This method, together with a voltage-sensing dark fluorescence quencher, disperse orange 3 (D3), has been extensively tested and shown to lack adverse effects on neuronal properties (Alich et al., 2021). We used the dqGEVI for the first time in a human *in vitro* disease-modeling study involving iPSC-derived sensory neurons.

Chronic pain is the leading cause of disability worldwide (Rice et al., 2016) with a prevalence of up to 40% (Tsang et al., 2008; Johannes et al., 2010) and a comorbidity with a number of highly widespread disorders, such as cancer, diabetes and HIV-induced peripheral neuropathy. As a large percentage of patients report their pain treatment as unsatisfactory, new therapeutics and novel cellular models are urgently needed. Using dqGEVI we monitored the spontaneous activity of hiPSC-derived sensory neurons (hiPSCdSN) at physiological temperatures. We applied this approach to hiPSCdSN derived from patients with inherited erythromelalgia (EM), a genetic chronic pain disorder with recurrent attacks of pain, swelling and redness of distal extremities. We show that dqGEVI enables the detection of significant differences in bursting behavior when compared to hiPSCdSNs from a non-EM cell line, a phenotype that has not been detected in a previous patch-clamp based study using the same cell line (Cao et al., 2016). Moreover, as in patients, where pain attacks can be triggered by mild increases in ambient temperature, dqGEVI enabled visualization of hyperexcitability in EM patient neurons upon temperature increase, a response absent in cells derived from the non-EM cell line.

To illustrate the general applicability of our approach we also recorded human iPSC-derived forward programmed GABA- and glutamatergic forebrain neurons termed iGABANs and iGlutNs, respectively (Peitz et al., 2020). In co-cultures of iGABANs and iGlutNs we observed spontaneous synchronous bursting behavior. This promising finding suggests that the dqGEVI will be broadly applicable to various other disease models such as Alzheimer’s and Parkinson’s disease, epilepsy and schizophrenia, conditions associated with disturbances of neuronal activity and synchrony (Uhlhaas and Singer, 2006).

## 2. Materials and methods

### 2.1. AAV production

The tetracycline operon system (“Tetbow” principle) (Sakaguchi et al., 2018) was used to enhance expression of GPI-eGFP, the genetically encoded component of the dqGEVI in iPSC-derived neurons. The template plasmid (pCAG Arc-eGFP-GPI) encoding GPI-eGFP was constructed by VectorBuilder, all other plasmids were purchased from Addgene. The coding region was subcloned to the pAAV-TRE-tdTomato-WPRE plasmid (Addgene ID: 104112) using the EcoRI and HindIII cut sites to give rise to the pAAV-TRE-GPI-eGFP-WPRE construct. This, and the pAAV-Syn1-tTA (Addgene ID: 104109) plasmids were used to produce recombinant adeno-associated viruses (rAAVs; mosaic “pseudotype” of AAV1 and AAV2) as described in our previous work (Alich et al., 2021) and originally by Hauck and Chen (2003). Functional titers (3.67 × 10e9 TU/ml for the TRE-GPI-eGFP-WPRE vector, and 5.81 × 10e9 TU/ml for the Syn1-tTA vector) were measured using *in vitro* limiting dilution assay.

### 2.2. hiPSC cultures

This study employed the wild type hiPSC-line UKBi013-A^1^ and the patient hiPSC-line RCi001-A^2^. The use of hiPSC-lines was approved by the Ethics Committee of the Medical Faculty of the University of Bonn (approval number 275/08), and informed consent was obtained from the donors. hiPSCs were cultured in StemMACS iPS-Brew (Miltenyi Biotec) and split with EDTA during maintenance. Quality control of hiPSCs for pluripotency (flow cytometric analysis of Tra1-60 expression) and genomic integrity (SNP analysis) was performed on a routine basis before *in vitro* differentiation as previously described (Elanzew et al., 2020).

### 2.3. Differentiation of hiPSCs into sensory neurons

Differentiation of hiPSCs into sensory neurons was performed following the protocol from Chambers et al. (2012) with slight modifications: In detail single cell suspensions of hiPSCs were seeded at suitable densities in StemMAC iPS-Brew in the presence of 10 μM ROCK inhibitor Y-27632 on Geltrex coated T175-flasks at day 1. Initial seeding densities needed to be determined for each cell line and can range between 3 × 10^5^ and 10 × 10^5^ cells/cm^2^. A total of 24 h after plating medium was changed to differentiation medium. Two basal media were used during differentiation: Medium 1 consists of Knock out DMEM with 20% Knock Out Serum Replacement, 2 mM (1x) GlutaMAX, 100 μM (1x) NEAA and 0.02 mM 2-mercaptoethanol. Medium 2 consists of Neurobasal Media supplemented with 1% N2 supplement, 2% B27 supplement, 2 mM (1x) GlutaMAX, and 0.02 mM 2-Mercaptoethanol. Between day 0 and day 3 cells were kept in Medium 1, from day 4 to 5 cells were kept in 75% Medium 1 and 25% Medium 2, for day 6 cells were kept in 50% Medium 1 and 50% Medium 2, from day 7 to 9 cells were kept in 25% Medium 1 and 75% Medium 2 and from day 10 to 14 differentiating cells were kept in 100% Medium 2. Neuronal differentiation was induced by dual-SMAD inhibition. 100 nM LDN 193189 and 10 μM SB 431542 were added from day 0 to day 6. To specify the differentiating cells into sensory neurons 3 μM CHIR 99021, 10 μM SU 5402 and 10 μM DAPT were added to the culture from day 3 to day 14. At day 14 of differentiation, cells were dissociated to single cells by treatment with accutase for 45–60 min, centrifuged at 400 × g for 10 min, resuspended in cold CryoStor CS10 freezing medium and frozen at −80^°^C in freezing boxes. Frozen cells were transferred to a liquid nitrogen tank for long-term storage after 24 h.

Frozen hiPSC-derived sensory neurons were thawed, counted and plated at a density of 40,000 cells per 12 mm coverslip (Neuvitro Corporation, GG-12-PLO-Laminin, Camas, WA, USA) in maturation media supplemented with 10 μM ROCK inhibitor Y-27632. Coverslips were pre-coated with 360 μg/ml Geltrex for 1 h at room temperature. Maturation medium consists of Neurobasal A Medium supplemented with 1% N2 supplement, 2% B27 supplement, 2 mM (1x) GlutaMAX, 0.02 mM 2-mercaptoethanol, 12 μg/ml gentamicin, 200 μM ascorbic acid, 0.1 μg/ml human recombinant laminin (BioLamina, LN521), 10 ng/ml GDNF, 10 ng/ml BDNF, 10 ng/ml NGF, and 10 ng/ml NT3. Medium was changed after 24 h to remove ROCK inhibitor. After 3 days cells were treated with 1 μg/ml Mitomycin C (Sigma Aldrich, M4287) for 2 h at 37^°^C to inactivate proliferative cells. Recombinant adeno-associated viruses (rAAVs) were added on day 6 after thawing (2 μl AAV-Syn tTA; 2 μl AAV- TRE-GPI-eGFP-WPRE) and were incubated with the cells for 1 week without media change. Afterwards, the medium was changed twice per week. Cells were matured for at least 6 weeks prior to functional analysis.

### 2.4. Differentiation of hiPSCs into iGlutNs and iGABANs

Induced forebrain glutamatergic neurons (iGlutNs) and GABAergic neurons (iGABANs) were generated by controlled overexpression of NGN2 and ASCL + DLX2 in hiPSCs (lines iLB-C14-s11-NGN2 and iLB-C-133-s4-APD) as described (Rhee et al., 2019; Peitz et al., 2020). Mixed cultures comprising 80% iGlutNs and 20% iGABANs (both 8 days after transgene induction) were plated at a density of 1,000 cells/mm^2^ in 24-well plates containing Matrigel-coated glass cover slips. The following day 50% of the medium was changed to fresh pre-warmed NBB27 medium (Neurobasal medium, 1x B27 supplement, 1x GlutaMax, 10 ng/ml BDNF, 2–4 μg/ml laminin and 1 μg/ml doxycycline). After 1 h rAAVs were added and incubated overnight after which the medium was changed to 0.5 ml/well of fresh pre-warmed astrocyte-conditioned NBB27. For the following 3 days medium was changed daily, and on day 4 post transduction mouse astrocytes generated from P1 pups were added in NBB27/FBS (Neurobasal medium, 1x B27 supplement, 1x GlutaMax, 10 ng/ml BDNF and 0.5% heat-inactivated FBS) medium to stabilize neuronal maturation. Medium changes with NBB27/FBS were done twice a week until the start of measurements.

### 2.5. Confocal imaging

To demonstrate plasma membrane targeting of the GPI-eGFP fusion protein, images of transgene-expressing neurons were taken using confocal laser scanning microscopy (Fluoview 1000, Olympus) at 40 × magnification. Cells were fixed with 4% paraformaldehyde for 10 min and coated with DAPI-Vectashield mounting medium (Vector Laboratories) prior to imaging.

### 2.6. Immunofluorescence characterization of hiPSC-derived sensory neurons

Cells fixed in 4% PBS-buffered paraformaldehyde for 10 min were permeabilized with 0.3% Triton X-100 in PBS for 15 min at room temperature, to stain for intracellular proteins. After washing, cells were incubated for 1 h at room temperature in blocking buffer (PBS, 5% normal donkey serum (NDS), 0.1% Triton X-100). Primary antibodies were incubated overnight at 4^°^C in PBS supplemented with 1% NDS and 0.1% Triton-X100 (mouse-anti-Islet1, Abcam, Cambridge, UK ab86501, 1:100; mouse-anti-Peripherin, Santa Cruz Biotechnology, Dallas, USA, sc-377093, 1:200; rabbit-anti-Brn3a, Merck, Darmstadt, Germany, AB5945, 1:200; rabbit-anti-Nav1.8, Alomone Labs, Jerusalem, Israel, ASC-016, 1:200). Cells were washed twice with PBS and incubated with secondary antibodies for 1 h at room temperature. Secondary antibodies were diluted 1:500 in PBS supplemented with 1% NDS and 0.1% Triton-X100 (donkey-anti-rabbit IgG-Alexa Fluor^®^ 488, Thermo Fisher, Waltham, MA, USA, A-21206; donkey-anti-mouse IgG-Alexa Fluor^®^ 594, Thermo Fisher; donkey-anti-goat IgG-Alexa Fluor^®^ 647, Thermo Fisher, A-21203). Extracellular markers were stained accordingly, but without the permeabilization step and without Triton-X100 in any of the incubation and blocking solutions (mouse-anti-Nav1.7, Abcam, ab85015, 1:100; goat-anti-hRet, R&D Systems, Minneapolis, MN, USA, AF1485, 1:200). After washing, coverslips were mounted on high precision cover glasses using Fluoromount-G with DAPI (Invitrogen, 00-4959-52). Images were taken with ZEISS Axio Imager Z1 equipped with an Apotome 1.0.

### 2.7. Genomic DNA isolation and Sanger sequencing

Genomic DNA was isolated of wild type and patient hiPSC using Qiagen DNeasy Blood and Tissue Kit. The DNA section harboring the SCN9A V400M mutation in the patient cell line was amplified *via* PCR, using the NEB Q5^®^ High-Fidelity 2X Master Mix with the 5′-ATT TCC ATT TTT CCC TAG ACG CTG-3′ forward, and the 5′-TAC CTC AGC TTC TTC TTG CTC TTT-3′ reverse, primers (Cao et al., 2016). Amplicons were purified and analyzed by Sanger sequencing.

### 2.8. Optical recordings

Reagents for electrophysiological and optical experiments were purchased from Sigma (St. Louis, MO, USA) unless indicated otherwise. Optical experiments were conducted using an Olympus BX61WI microscope (Olympus Corporation, Tokyo, Japan) equipped with epifluorescence and DIC. A scientific complementary metal-oxide-semiconductor (sCMOS) camera (Prime 95B, Teledyne Photometrics, Tucson, AZ, USA) was used to visualize neurons and to verify fluorescence. GPI-eGFP expressing cultured hiPSC-derived sensory neurons 8–10 weeks, and cortical neurons 10–13 weeks after viral transduction were transferred to a modified submerged chamber (Hill and Greenfield, 2011) and perfused with HEPES-buffered ACSF (3 ml/min at 37 ± 0.5^°^C, in mM): 135 mM NaCl, 4.7 mM KCl,1 mM CaCl2, 1 mM MgCl2, 10 mM Hepes, and 10 mM glucose (pH was adjusted to 7.4 with NaOH). Recordings were started after a ∼5 min preincubation period with ACSF containing of the diazobenzene dye D3 (2 μM) in 0.02% DMSO. The sCMOS camera was controlled by μManager software (Edelstein et al., 2014). Expression of GPI-eGFP was verified using epifluorescence. Excitation illumination (470 nm) at 3–5 mWcm^–2^ was provided with a custom made light source (parts from Thorlabs Inc, Newton, NJ, USA with a Luxeon Rebel 470 nm LED LXML-PB01-0040) that was driven by a custom made TTL switched stable current source. The excitation light as well as the collected fluorescence were filtered using a FITC filter set (Ex: HQ480/40x; Di: Q505LP; Em: HQ535/50 m, Chroma Technology Corp., Bellow Falls, VT, USA). For optical voltage imaging, frame rates of 976 FPS were achieved using smaller field of view [regions of interest (ROIs)] with 52 pixels in the vertical plane and variable pixel in the horizontal plane. Camera was used in 12 bit sensitivity mode resulting in a gain of 0.67 e-/ADU. Exposure time was 1 ms. No binning was applied. Imaging traces were usually recorded for 10 s.

For heat sensitivity experiments hiPSC - derived sensory neurons were heated to 40^°^C with a custom-made perfusion system. Temperature was calibrated before each recording and controlled for deviations from set temperature of 40^°^C before and after each recording. Imaging traces were recorded for 30 s. In some experiments, the Nav1.7 Inhibitor (PF-05089771, Tocris Cat. No. 5931, Batch No: 1A/225855) was applied to the cells after heating to 40^°^C (Supplementary Figure 3B).

For Nav1.7 specificity experiments the Nav1.7 Inhibitor (PF-05089771, end concentration 60 and 110 nM) was added to the ACSF and active cells were perfused (3 ml/min at 37 ± 0.5^°^C) with the custom-made perfusion system.

For all experiments a 100 mM stock solution was prepared in DMSO and stored at −20^°^C.

### 2.9. Image processing pipeline

The video frames were processed using a custom-written app in MATLAB (MathWorks, USA). The stack of frames was loaded as grayscale images. An image file from the whole stack was selected as a reference frame for further image processing and finding the ROIs of the cells. The reference frame was filtered using a 2D Gaussian kernel with a standard deviation of one for removing noise. The grayscale image was binarized using the adaptive threshold method. The cells in the field of view were segmented after applying morphological image processing operations and then the reference ROIs were automatically generated. The mean intensity in the ROI of each frame in the whole stack was calculated and used as the fluorescence signal of the corresponding cell. The extracted intensity for each frame was plotted to obtain the fluorescence signal in the time domain. A linear fit was applied to the fluorescence trace to detrend a small drift caused by the camera. Then, the intensity values were converted into ΔF/F (%) scale. Spikes were detected using a threshold value of 3–5 standard deviation above the mean (Supplementary Figure 2).

### 2.10. Patch-clamp experiments

For electrophysiological recordings whole-cell patch clamp recordings were amplified using a Multiclamp 700B amplifier (Molecular Devices, Sunnyvale, USA), low-pass filtered at 10 kHz and digitized at 50 kHz with a NI USB-6341 (National Instruments, Austin, TX, United States) controlled by Strathclyde Electrophysiology Software WinWCP (John Dempster, University of Strathclyde, Glasgow, UK). Data were stored on a hard disk for offline analyses. Pipettes were pulled from borosilicate glass (King Precision Glass, Inc., Claremont, CA, USA) using a DMZ Zeitz puller (Zeitz-Instruments, Martinsried, Germany). Patch pipettes had resistances of 3–5 MΩ and contained (in mM): 135 K-Gluconate, 5 KCl, 10 HEPES, 0.1 ethylene glycol-bis (2-aminoethylether)-N,N,N′,N′-tetraacetic acid (EGTA), 1 MgCl_2_, 3 MgATP, 0.2 Na_2_ATP at pH 7.2.

## 3. Results

### 3.1. Expression of dqGEVI and optical detection of spontaneous spiking activity in hiPSC-derived sensory neurons

**FIGURE 1:**
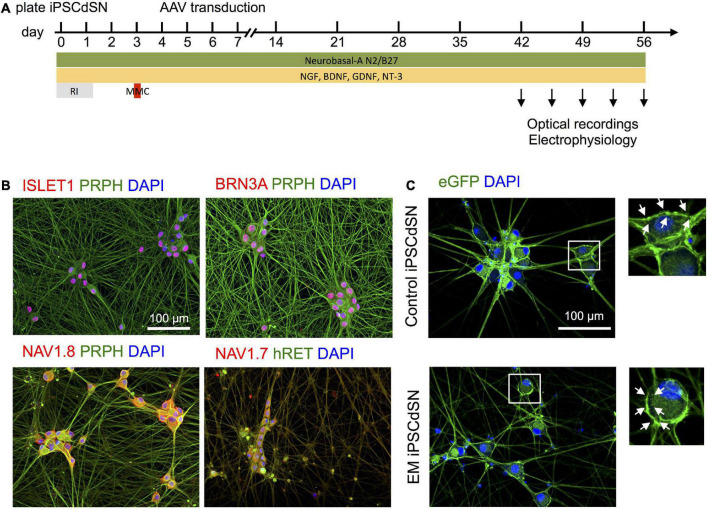
**(A)** *In vitro* cultivation and adeno-associated viruse (AAV) transduction paradigm for human induced pluripotent stem cell (hiPSC)-derived sensory neurons. RI, ROCK-Inhibitor, MMC: Mitomycin C. **(B)** Human induced pluripotent stem cell-derived sensory neuron (hiPSCdSN) express typical sensory neural marker, such as ISLET1, BRN3A, and PRPH, as well as peripheral sodium channels Nav1.7 and Nav1.8. **(C)** Dark quencher hybrid genetically encoded voltage indicator (dqGEVI) specifically labels the sensory neuronal cell membrane. EM, erythromelalgia.

Human induced pluripotent stem cells from a healthy donor with a wild type *SCN9A* gene (UKBi013-A) and an EM-patient, suffering from a gain-of-function mutation in the *SCN9A* gene (RCi001A) previously characterized as “EM3” in Cao et al. (2016) were differentiated into immature sensory neurons within 14 days using a small-molecule based protocol and cryopreserved (Supplementary Figure 1). Cells are referred to as EM patient hiPSCdSNs and control hiPSCdSNs (for EM patient and control cells, respectively). The dqGEVI genes were delivered *via* AAV-based transduction (see section “2 Materials and methods”), 6 days after thawing and plating of the hiPSCdSN (Figure 1A). Analysis of marker expression and optical recordings of hiPSCdSNs were performed after 6–8 weeks culture in growth factor containing medium. At that time hiPSCdSN showed clustering, forming ganglion-like structures and a dense neuronal network. Immunofluorescence analysis revealed expression of classic sensory neuron markers including BRN3A, ISLET1, PRPH, and hRET, as well as the peripheral neuron specific voltage gated sodium channels Nav1.7 and Nav1.8 in virtually all neurons after 8 weeks of *in vitro* culture (Figure 1B). Expression of dqGEVI was confirmed by detection of native eGFP expression (Figure 1C). EGFP expression was robust and restricted to the plasma membrane at the soma with only a few of the cells expressing intracellular fluorescence, most likely at the ER membrane. There were no intracellular punctae as described elsewhere with other voltage indicators (Kiskinis et al., 2018).

Expression of dqGEVI does not affect passive or active membrane properties of primary mouse neurons (Alich et al., 2021). To verify these observations in hiPSCdSNs we performed manual patch-clamp experiments in cells transduced with dqGEVI and PBS as control. We did not observe differences in input resistance (R_*in*_), membrane capacitance (C_*m*_), time constant, and resting membrane potential (V_*rest*_) after expression of dqGEVI in EM patient hiPSCdSN (pt) and control hiPSCdSNs (Ctrl) (R_*in*_: pt: dqGEVI: 105.5 ± 11.34 MΩ (*n* = 11), PBS: 80.8 ± 9.99 MΩ (*n* = 11), Ctrl: dqGEVI: 61.1 ± 4.2 MΩ (*n* = 10), PBS: 48.3 ± 5.1 MΩ (*n* = 13), *p* = 0.27; C_*m*_: pt: dqGEVI: 128 ± 10 pF (*n* = 11), PBS: 167 ± 26 pF (*n* = 11), Ctrl: dqGEVI: 169 ± 11 pF (*n* = 10), PBS: 231 ± 31 pF (*n* = 13), *p* = 0.15; time constant: pt: dqGEVI: 0.012 ± 0.0004 ms (*n* = 11), PBS: 0.011 ± 0.0006 ms (*n* = 11), Ctrl: dqGEVI: 0.010 ± 0.0007 ms (*n* = 10), PBS: 0.010 ± 0.0013 ms (*n* = 13), *p* = 0.06; V_*rest*_: pt: dqGEVI: -53.1 ± 2.44 mV (*n* = 11), PBS: -58.8 ± 1.3 mV (*n* = 11), Ctrl: dqGEVI: -52.5 ± 1.41 mV (*n* = 10), PBS: -55.65 ± 1.2 mV (*n* = 13), *p* = 0.06; Kruskal-Wallis test, significance level α = 0.05) indicating that the integrity of the plasma membrane is fully preserved. Additionally, as we did not observe a difference in these parameters between control hiPSCdSN and EM patient hiPSCdSN, we conclude that the differences in firing patterns between control hiPSCdSN and EM patient hiPSCdSN were not due to differences in passive membrane characteristics.

### 3.2. A hyperactive phenotype in EM patient hiPSCdSNs

**FIGURE 2:**
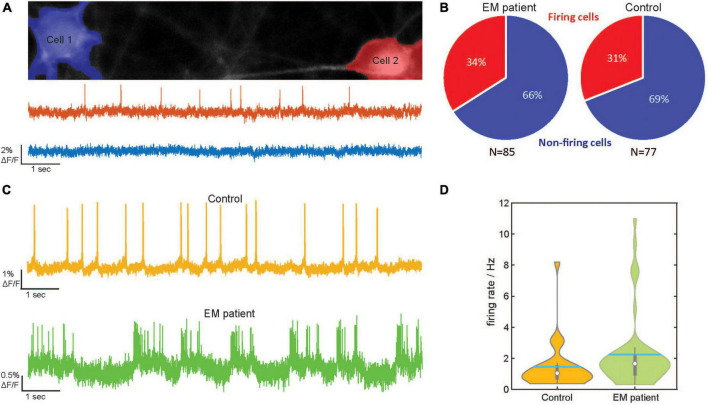
Optical detection of spontaneous spiking in human induced pluripotent stem cell -derived sensory neurons (hiPSC–SNs) from an erythromelalgia (EM) patient and control. **(A)** Top: Images of two (Cell 1 in blue and Cell 2 in red) control hiPSC–SNs expressing dark quencher hybrid genetically encoded voltage indicator (dqGEVI). Somata were detected with an automated soma detection and evaluation procedure. Bottom: Raw imaging traces of cell 1 (blue) and cell 2 (red). **(B)** Pie-graphs showing the percentage of spontaneously firing cells from EM patient (Total *N* = 85, active *N* = 29) and control (Total *N* = 77, active *N* = 24). **(C)** Representative raw imaging traces of spontaneous firing behavior of hiPSC-SNs from the EM patient (bottom) and control (top). **(D)** Violin plots of the firing rate distribution in the EM patient human induced pluripotent stem cell-derived sensory neurons (hiPSCdSNs) (*N* = 45) and the control hiPSCdSNs (*N* = 27). The thin vertical black line indicates the 95% confidence interval. The thick vertical black line indicates the interquartile range. The median is indicated by the white dot. Spiking frequency is significantly higher in hiPSCdSNs from EM patients (Wilcoxon Rank test, WRT, *p* = 0.03).

We assessed the number of spontaneously active cells in the EM patient hiPSCdSNs and control hiPSCdSNs using an automated cell finding and evaluation pipeline (described in “2 Materials and methods”). The pipeline provides algorithms to automatically identify and segment neurons (Figure 2A, top), to extract the fluorescent signals and to detect spikes (Figure 2A, bottom). Other approaches for voltage imaging analysis make use of image processing algorithms (Kiskinis et al., 2018; Cai et al., 2021) to extract the fluorescent signal. Notably, in our analyses data were not filtered, smoothed or averaged for analysis as the single raw optical traces possessed sufficient signal to noise ratio for accurate spike detection (Figures 2A, C).

Analysis of the imaging traces revealed that in both groups about a third of the cells showed spontaneous activity (EM patient: 34% active cells of total *n* = 85; control 31% active cells of total *n* = 77; Figure 2B). These results are partially in line with the findings from Cao et al. (2016), who detected 35% spontaneously active EM neurons. However, in contrast to our data, only 0–14% of their control neurons showed signs of spontaneous activity. This discrepancy could be due to intrinsic heterogeneity among the hiPSCdSN preparations or variations among healthy donors. Disturbances of the cell interior dialysis of the intracellular solution by the patch-clamp technique used in their study may also account for these differences. The fraction of active EM patient hiPSCdSN, however, revealed a significantly higher firing rate in the EM patient hiPSCdSNs (mean spiking frequency 2.18 ± 0.35 s^–1^, median 1.56) compared to the control hiPSCdSNs (mean spiking frequency 1.46 ± 0.3 s^–1^, median 1.04; *p* = 0.03 Wilcoxon rank-sum test) (Figures 2C, D). Thus, EM patient sensory neurons show a hyperactive phenotype, a finding which is in line with previous observations and can be attributed to the gain-of-function mutation *SCN9A* gene encoding the voltage-gated sodium channel subunit alpha Nav1.7 (Cao et al., 2016). The specificity of this phenotype was further confirmed by the application of the specific Nav1.7 channel blocker PF-05089771, which at 60 and 110 nM concentrations blocked AP firing of the patient hiPSCdSNs (data not shown).

### 3.3. Burst AP firing in EM patient hiPSCdSNs

The temporal pattern of AP firing is essential for sensory information (pain) transmission, as spikes that occur in a bursty pattern (burst spikes) can be transmitted across synapses more reliably than spikes occurring in an irregular (sporadic spikes) pattern (Lisman, 1997).

**FIGURE 3:**
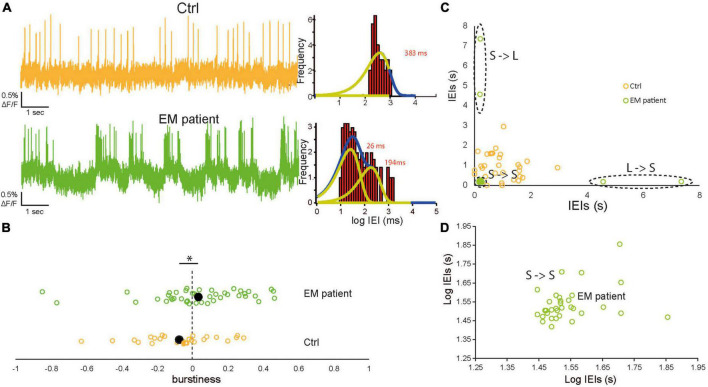
Increased spontaneous bursting discharge in erythromelalgia (EM) patientdSNs. **(A)** Left: Representative raw imaging traces of control human induced pluripotent stem cell-derived sensory neurons (hiPSCdSNs) (top, orange) showing a sporadic firing pattern and EM patient hiPSCdSNs (bottom, green) showing a clear bursting pattern. Right: Log-binned Interevent interval (IEI) histograms of the example EM patient hiPSCdSNs trace exhibited two distributions that were well fit with multiple exponential distributions whereas control hiPSCdSNs were well fit with a single exponential. Yellow lines indicate exponential fits. Blue lines indicate the sum of the fits. **(B)** Comparison of the burstiness of EM patient hiPSCdSNs and control hiPSCdSNs. Filled black dots indicate the average values. Burstiness is significantly higher in EM patient hiPSCdSNs (*EM patientdSNs N* = 45, control hiPSCdSNs *N* = 27; WRT *p = 0.04). **(C)** Plot of the shifted IEIs for single cell examples of EM patient hiPSCdSNs and control hiPSCdSNs. Dotted black lines indicate clusters of short followed by short (S->S) Short followed by long (S->L) and L->S in the EM patient group. **(D)** Enlargement of the S->S cluster in **(C)** on a log scale.

We therefore analyzed the bursting pattern of AP firing of the EM patient and control hiPSCdSNs on cells showing spontaneous activity. We found that control hiPSCdSNs discharge in sporadic fashion, while EM patient hiPSCdSNs discharge in a bursting pattern (Figure 3A, left shows example traces). The log-binned histograms of the time difference between the spikes, the inter-event intervals (IEIs; Figure 3A, right) show that the sporadically firing control cells can be fitted with a single exponential, whereas the bursting EM cells are well-fitted with two exponentials, representing the intra-burst intervals between APs (left peak) and the inter-burst intervals between two bursts (right peak). Exponential fits were performed on all cells with >20 APs. In EM patient hiPSCdSNs 15 out of 30 cells exhibited two distributions (average tau values: intra-burst intervals: 165 ± 38 ms; inter-burst intervals 1,750 ± 377 ms), the remaining 15 cells exhibited a single distribution (average tau values for single fit: 852 ± 220 ms). In control hiPSCdSNs 6 out of 24 cells exhibited two distributions (average tau values: intra-burst intervals: 313 ± 111 ms; inter-burst intervals 1,775 ± 603 ms), the remaining 18 cells exhibited a single distribution (average tau values for single fit: 974 ± 207 ms). There was no difference between intra-burst intervals and inter-burst intervals from EM patient hiPSCdSNs and control hiPSCdSNs in the cells exhibiting two distributions (intra-burst intervals *p* = 0.028; inter-burst intervals *p* = 0.87, Wilcoxon rank-sum test) and in the cells exhibiting a single distribution (*p* = 0.69, Wilcoxon rank-sum test) indicating that the quality of the bursts is not different between groups. To quantify these observations we categorized the cells into “bursters” and sporadically firing cells (Table 1). A “burster” was defined as having an epoch where at least one burst occurred in which a “burst” is defined as a cluster of spikes from a single neuron that fires with a higher rate than at a previous time (Lobb, 2014).

**TABLE 1:** Number of bursting versus sporadically firing cells in erythromelalgia (EM) patient versus control human induced pluripotent stem cell-derived sensory neurons (hiPSCdSNs).

	Bursters	Sporadically firing cells	Marginal row totals
Control	4	26	30
EM patient	23	44	67
Total	27	70	97 (grand total)

Fischer’s exact test *p* = 0.049.

The results reveal that the fraction of bursters is significantly higher in EM patient hiPSCdSNs compared to control hiPSCdSNs (EM patient: total *n* = 67 Ctrl: total *n* = 30, Fisher’s exact test *p* = 0.049) (Table 1). To further support our statement on the difference between bursting vs. non-bursting EM patient hiPSCdSNs vs. control hiPSCdSNs we performed a bootstrap Chi-square analysis on 30 randomly picked cells from the patients and the 30 Ctrl with 10,000 iterations without replications. The mean *p*-value from all iterations is 0.0179.

We also characterized the firing patterns of the cells using a continuous measure of burstiness. The burstiness parameter ***B*** was calculated as in previous papers (Goh and Barabási, 2008; Schleiss and Smith, 2016; Chen et al., 2018):


B=(σ-μ)/(σ+μ)

where σ and μ denote the standard deviation and the mean of inter-event intervals (IEIs), respectively. The magnitude of B (−1, 1) correlates with the cell’s burstiness with B = 1 indicating a bursty signal, B = 0 a neutral signal (Poisson process), where the IEIs follow an exponential distribution, and B = −1 indicating a completely regular (periodic) signal. We observed a significantly higher burstiness in the EM patient hiPSCdSNs compared to control hiPSCdSNs (Wilcoxon rank-sum test, *p* = 0.02) (Figure 3B).

Another measure of the burstiness of a cell is the accumulation of consecutive short IEIs, which represent the spikes within a burst. To categorize the IEIs of each cell into consecutive short (long) followed by short (long) (S->S, L->L), we plotted the IEIs of a cell against itself shifted by one and clustered the data using the k-means method with k = 3 for both groups (Figures 3C, D). The quality of the clustering was controlled by calculating the silhouette index for the clustering with k = 3. All values were >0.5. Average silhouette indexes were 0.70. ± 0.15 for EM neurons and 0.73 ± 0.14 for controls, indicative of a qualitatively median to good clustering. We counted the S->S intervals in the cells where a clustering was enabled by the presence of three obvious clusters. Cells with silhouette indices ≥0.5 were included in the analysis. In the EM patient group an average of 38.5 ± 4.7 S->S were counted compared to 16.1 ± 4.3 S->S intervals in the control. Statistical comparison of the data shows a significant difference (Wilcoxon rank-sum test, *p* = 0.002).

### 3.4. Elevated heat sensitivity in EM patient hiPSCdSNs

**FIGURE 4:**
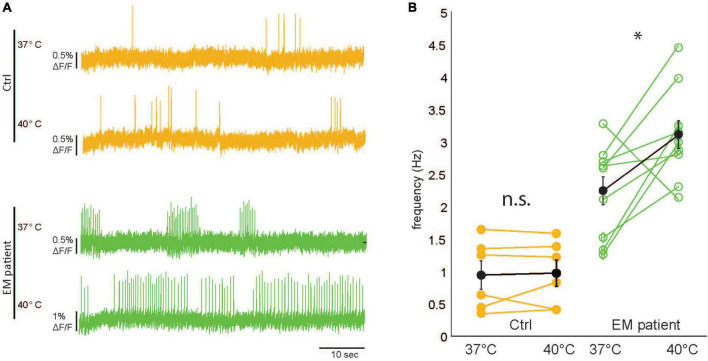
Erythromelalgia (EM) patient human induced pluripotent stem cell–derived sensory neurons (hiPSC–SNs) are hypersensitive to mild increases in ambient temperature. **(A)** Representative raw imaging traces of control hiPSC-SNs (top, orange) and EM patient–SNs (bottom, green) at 37 and 40^°^C. **(B)** Comparison of spike frequency filled black dots indicate average values. Error bars indicate SEM. [*EM patientdSNs N* = 10, control human induced pluripotent stem cell-derived sensory neurons (hiPSCdSNs) *N* = 6, Wilcoxon rank-sumtest, control: *p = 0.87, EM patient: *p = 0.027].

Recurrent attacks of pain and swelling in the distal extremities characteristic for inherited erythromelalgia are, amongst others, triggered by mild increases in ambient temperature. To elucidate if the present model can mimic the elevated heat sensitivity observed in EM patients, we increased the ambient temperature from 37^°^C (recording temperature) to 40^°^C (example traces shown in Figure 4A) in EM patient and control cells. The baseline frequencies in the EM patient hiPSCdSNs were significantly higher as compared to control hiPSCdSNs (*p* = 0.005, Wilcoxon rank-sum test), which reflects the increased excitability described in Figure 2. We observed a significant increase in firing frequency in the EM patient hiPSCdSNs () but not in the control hiPSCdSNs [EM patient hiPSCdSNs *n* = 10, 2.25 ± 0.21 to 3.12Hz ± 0.22 Hz at 40^°^C, *p* = 0.03; control hiPSCdSNs *n* = 6, 0.96 ± 0.22 to 0.99 ± 0.21 Hz at 40^°^C, *p* = 0.87, Wilcoxon signed-rank test (paired difference test), all errors are indicated as ± SEM Figure 4B. These observations are in line with the findings of Cao et al. (2016), further reinforcing the role of Nav1.7 channels in the elevated heat sensitivity observed in EM patients.

### 3.5. Exploration of dqGEVI for optical recording of hiPSC-derived forebrain neurons

To demonstrate a broader applicability of the dqGEVI platform for hiPSC-based neurological and neuropsychiatric disease modeling we expressed dqGEVI in mixed cultures of human hiPSC-derived forebrain neurons. The synchrony of neuronal firing is of major importance for information processing in the brain, and abnormal neuronal synchronization has been found in diverse neurological and neuropsychiatric diseases such as schizophrenia, epilepsy, autism spectrum disorder, Alzheimer’s and Parkinson’s reviewed in Uhlhaas and Singer (2006).

To generate defined populations of forebrain neurons we employed forward programming of iPSCs via overexpression of the transcription factors NGN2 and ASCL1 + DLX2 to generate induced glutamatergic (iGlutNs) and GABAergic neurons (iGABANs), respectively (Zhang et al., 2013; Yang et al., 2017). To ensure highly controlled and stable transgene expression, we used targeted insertion of doxycyclin-inducible transgene cassettes into the AAVS1 “safe harbor” locus as described (Rhee et al., 2019; Peitz et al., 2020).

**FIGURE 5:**
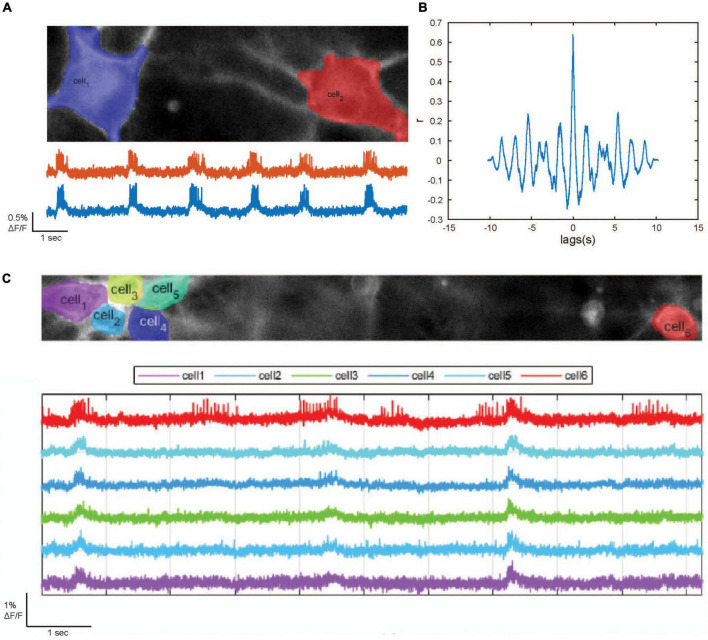
Optical detection of epileptogenic bursting behavior in human induced pluripotent stem cell (hiPSC)-derived forebrain neurons [induced forebrain glutamatergic neurons (iGluN) (80%) and GABAergic neurons (iGABAN) (20%)]. **(A)** Top: Automated detection of the somata of two example hiPSC-derived iGluN iGABAN neurons. **(B)** Cross-correlation between the fluorescence traces of two example neurons. **(C)** Upscaling of the dark quencher hybrid genetically encoded voltage indicator (dqGEVI) imaging system. Top: Automated detection of the somata of six hiPSC-derived forebrain neurons. Bottom: respective fluorescence traces.

For optical recording we used mixed cultures composed of 80% NGN2-induced and 20% ASCL1/DLX2-induced neurons. Upon transduction with the dqGEVI-expressing AAVs we imaged the spontaneous activity of these cultures (Figure 5A) at 37^°^C (batch of *n* = 48 neurons, mean firing frequency 2.57 s^–1^) and calculated the correlation coefficient of the optical sampling points (Figure 5B) as a measure of synchronized activity between pairs of neurons using a sample of 18 pairs. The mean correlation coefficient was 0.436 ± 0.036, indicating a high degree of synchrony between neuronal pairs.

To demonstrate that the dqGEVI system can be scaled up toward a high throughput screening platform we enlarged the field of view (FOV) to 66 × 664 pixels to observe the synchronization between more cells. We were able to visualize the synchronous activity of six neurons in parallel (Figure 5C). The number of neurons in the FOV is thereby solely restricted by the capacity of the recording computer. The FOV can, without reservations in terms of sampling rate, be extended to 1,200 pixels (pixel size 11× 11 μm) in the horizontal plane, and paired with an increase in cell density the number of cells in the FOV can be further increased.

These data indicate that the present dqGEVI approach can be used to record both PNS and CNS neurons and that this method can be extended from single neurons to neuronal cohorts.

## 4. Discussion

In this study we employed a dqGEVI optical recording method to assess the spontaneous firing activity of hiPSC-derived sensory and forebrain neurons. This method represents the least invasive and still most accurate approach for AP detection and resolution during neuronal activity. In contrast to other GEVIs, in the dqGEVI the genetically encoded part of the sensor, the fluorescent protein (eGFP), is attached to the outside of the plasma membrane using a glycosylphosphatidylinositol anchor (GPI anchor) instead of transmembrane domains used in most of the other GEVIs such as rhodopsin-based and voltage sensitive domain (VSD)-based approaches reviewed in Yang and St-Pierre (2016), Knöpfel and Song (2019), Mollinedo-Gajate et al. (2021). The insertion of GPI-eGFP into the plasma membrane and the incubation with the voltage-sensing dark quenching molecule D3 has been shown to leave the membrane properties of the cells unaffected both in primary mouse neurons (Alich et al., 2021) and in hiPSC-derived neurons (present study).

In whole-cell patch-clamp recordings a significant opening in the plasma membrane and dialysis of the cells interior with the internal pipette solution might disturb the intracellular mechanisms of the neuron (Pusch and Neher, 1988). Intracellular ion concentrations are unavoidably disturbed by the dialysis of the cell’s interior into the pipette and the pipette’s contents into the cell. In addition, non-physiological intracellular Ca^2+^ buffering and the interference with endogenous Ca^2+^ buffers in these recordings poses a significant problem (Nägerl and Mody, 1998; Nägerl et al., 2000), but regrettably receives little attention. The intracellular solutions used in the whole-cell patch-clamp recordings contain various amounts of exogenous Ca^2+^ buffers and the most recently used molecules for optical recordings, the GECI, are themselves Ca^2+^ buffers (McMahon and Jackson, 2018). As cytosolic Ca^2+^ regulates many intracellular processes, it greatly affects the behavior of neurons. Synaptically connected neurons, which form neuronal networks, use intracellular Ca^2+^ to regulate their membrane excitability and to control the network bursting pattern (Kudela et al., 2009) mainly by influencing Ca^2+^-dependent K^+^ channels. Therefore, Ca^2+^ buffering in various recording methods is a major impediment for observing the physiological behavior of neurons, particularly their endogenous burst firing patterns.

The method employed here stands in sharp contrast to the whole-cell patch-clamp approach used by Cao et al. (2016), who previously investigated hiPSC derived sensory neurons from EM patients. Using the non-invasive dqGEVI approach we were able to observe the undisturbed spontaneous AP firing behavior of hiPSC-derived sensory and cortical neurons and could detect differences in AP firing phenotypes between patient and control neurons. The increased AP firing frequency, which we observed in the patient group is in line with previous patch-clamp based studies exploring patient cells from EM patients and mouse Nav1.7 mutant dorsal root ganglion (DRG) neurons (Dib-Hajj et al., 2005; Cao et al., 2016). These studies explained the increase in AP firing rate through a hyperpolarizing shift in activation and a depolarizing shift in steady-state inactivation of Na^+^ channels thus lowering the threshold for single APs. In addition to these observations, the dqGEVI method revealed that the firing behavior in the EM patient-derived cells differs not only with regard to AP frequency, but also in their burst firing patterns.

A burst is a group of events that are separated by gaps that are all shorter than a critical time *t*_*c*_ and a gap between the bursts that is longer than *t*_*c*_. Various methods have been used to differentiate between within bursts and between bursts time gaps, and to minimize misclassifications (Colquhoun and Sakman, 1985; Lieberman and Mody, 1998).

Using several methods of burst classification, we found a significantly larger fraction of “bursters” in the patient group. This finding might shed light on the pathophysiological mechanism of EM. If neurons fire in bursts, the information transmission within neuronal networks is increased due to the increased probability of inducing postsynaptic neurons to fire APs (Lisman, 1997; Izhikevich et al., 2003). This is of particular relevance for pain transmission, as burst firing of the dorsal root ganglion (DRG) cells increases the likelihood for pain information transmission to the postsynaptic pain projection neuron.

Heat evokes intense burning pain in EM patients (Drenth and Waxman, 2007). This is mimicked in our model where we observed a marked increase in mean firing frequencies upon slight increases in ambient temperature in Nav1.7 mutant cells, whereas the mean firing rate in control hiPSCdSNs only showed a minimal increase as in EM hiPSCdSNs (Cao et al., 2016) and in rat DRG neurons (Yang et al., 2016). It is long known that the firing pattern and the electrical activity of neurons is affected by changes in ambient temperature (Hodgkin and Katz, 1949; Dalton and Hendrix, 1962; Frankenhaeuser and Moore, 1963; Burrows, 1989). These temperature changes influence membrane excitability by altering ion channels and active transporters. The effect of temperature on neuronal excitability, however, is bidirectional. Temperature increases can trigger activation of transient receptor potential (TRP) channels. Upon stimulation these channels produce “generator potentials,” which are small changes in voltage across the membrane. TRP channels are expressed in different subsets and at different expression levels thereby transducing thermal stimuli of different strength to produce these “generator potentials” (Vriens et al., 2014). Nav1.7 channels then act as amplifiers of these receptor potentials causing the cell to fire once the potential has reached a certain threshold (Waxman, 2006) thus contributing to an increase in excitability upon heating.

On the other hand, temperature increases can reduce input resistance by affecting potassium channels such as TREK2 and TRAAK, which are expressed in DRG neurons (Kang et al., 2005), thereby inhibiting APs firing and decreasing neuronal excitability. The minimal temperature effect on control neurons is thus likely to result from the balanced interaction between different channel types, TRP, K^+^ and Na^+^. We propose that the Nav1.7 gain of function mutation in the patient hiPSCdSNs shifts this balance toward a hyperexcitable phenotype as the receptor potentials are strongly amplified.

We observed 34% of spontaneously active cells in the EM patient group, which is in line with the findings from Cao et al. (2016), where their EM3 cells showed 35% active cells. A similar fraction of active cells, 31%, was detected in our control hiPSCdSNs. The comparable ratios of active versus non-active cells in the patient hiPSCdSNs and the control hiPSCdSNs may suggest that the fraction of active cells is independent of the sodium channel mutation and does not contribute to the EM phenotype.

We also demonstrate the usefulness of the optical voltage recordings in mixed cultures of glutamatergic and GABAergic forebrain neurons. Targeting gene expression to a defined subset of nerve cells using cell type specific promoters may refine the approach even further and enable deciphering the contribution of a particular neuronal subtype to a disease phenotype or to visualize differences in susceptibility toward pharmacological compounds. This strategy is, however, particularly challenging in hiPSC-derived neurons, because the currently available promoters are often leaky (resulting in non-specific transgene expression) and maintain a poor expression level, which is highly disadvantageous in voltage imaging. The bipartite expression system presented here provides a straight-forward solution for the latter problem. The weak, but neuron-specific Synapsin1 promoter maintains sufficient levels of the trans-activator protein to induce adequate levels of fluorescent protein expression *in trans* for imaging in the co-transduced cells. Following the same principle, other specific promoters such as the mGAD65 should be able to restrict high levels of voltage sensor expression to a population of cells, e.g., inhibitory neurons (Hoshino et al., 2021). Furthermore, diluting the trans-activator encoding rAAV also facilitates sparse, stochastic labeling of the transduced cells which can be beneficial for removing background fluorescence of overlapping cells when imaging subcellular compartments.

In addition to being suitable for the study of hyperexcitable phenotypes, as in the example of inherited EM, in future studies the dqGEVI approach may also distinguish between phenotypes associated with changes in hyperpolarizing currents elicited by mutations in potassium channels. As originally shown (Alich et al., 2021) dqGEVI is highly sensitive to both hyperpolarizing and depolarizing subthreshold potential changes. In view of the multitude of neuropsychiatric diseases associated with mutations in potassium channels reviewed in Imbrici et al. (2013) this opens the prospect to a high throughput optical screening method of compounds for the treatment of a multitude of neuropsychiatric disorders. As reprogramming and differentiation or direct conversion of somatic cells into induced neurons becomes more and more reliable and scalable, the analyzed cohorts will steadily increase, creating a demand for a fast system ready for high throughput analyzes. Upscaling the system to a 96-well format using preexisting photodiode-based plate readers or automated camera based systems (e.g., HAMAMATSU FDSS/μCELL functional drug screening system) will increase the throughput and enable fast phenotyping of newly identified disease-associated mutations, as well as compound screening.

## Data availability statement

The datasets analyzed for this study can be found in the Sciebo cloud storage of the University of Bonn (https://uni-bonn.sciebo.de/s/6XN3D3eDLWraHoe).

## Ethics statement

The studies involving human participants were reviewed and approved by Ethics Committee of the Medical Faculty of the University of Bonn (approval number 275/08). The patients/participants provided their written informed consent to participate in this study.

## Author contributions

TA, OB, and IM: conceptualization. TA, PR, BS, ST, and MP: data curation. TA, PR, KG, MP, and IM: formal analysis. TA, PR, MP, BS, and ST: investigation. OB and IM: funding acquisition. IM: project administration. TA, KG, and IM: software. TA, PR, and IM: visualization. TA, PR, BS, and MP: writing—original draft. TA, OB, IM, BS, and PR: writing—review and editing. All authors contributed to the article and approved the submitted version.

## Footnotes

1
https://hpscreg.eu/cell-line/UKBi013-A

2
https://cells.ebisc.org/RCi001-A
