# Supplementary material for: Bringing to light the physiological and pathological firing patterns of human induced pluripotent stem cell-derived neurons using optical recordings
Source: Front Cell Neurosci. 2023 Jan 17;16:1039957. doi: 10.3389/fncel.2022.1039957 (PMC9887032; doi:10.3389/fncel.2022.1039957)
Supplement: Supplementary file 1 [file Data_Sheet_1.pdf]

# SUPPLEMENTARY INFORMATION

## Bringing to light the physiological and pathological firing patterns of human iPSC-derived neurons using optical recordings

**Alich TC ‡[,1], Röderer P ‡[,2,3], Szalontai B [**,1] , Golcuk K [1], Tariq S [2], Peitz M [2,4], Brüstle O [2] and Mody I [*,1,5]**

[1]Institute of Experimental Epileptology and Cognition Research, University of Bonn Medical Faculty & University Hospital Bonn, Venusberg-Campus 1, 53127 Bonn, Germany, therese.alich@ukbonn.de, balint.szalontai@uni-due.de, kgol@uni-bonn.de

[2]Institute of Reconstructive Neurobiology, University of Bonn Medical Faculty & University Hospital Bonn, Venusberg-Campus 1, 53127 Bonn, Germany, pascal.roederer@uni-bonn.de, tariqs3k@gmail.com, peitz@uni-bonn.de, brustle@uni-bonn.de

[3]LIFE & BRAIN GmbH, Cellomics Unit, Venusberg-Campus 1, 53127 Bonn, Germany

[4]Cell Programming Core Facility, University of Bonn Medical Faculty, Venusberg-Campus 1, 53127 Bonn, Germany

[5]Department of Neurology, Geffen School of Medicine at UCLA 635, Charles Young Drive South Los Angeles, CA 90095-733522/USA, mody@ucla.edu

‡ These authors contributed equally to this work

** Present address: Laboratory for Retinal Gene Therapy, Department of Ophthalmology, University Hospital Zürich, University of Zürich, Zürich, Switzerland

*** Correspondence:**
Istvan Mody
mody@ucla.edu

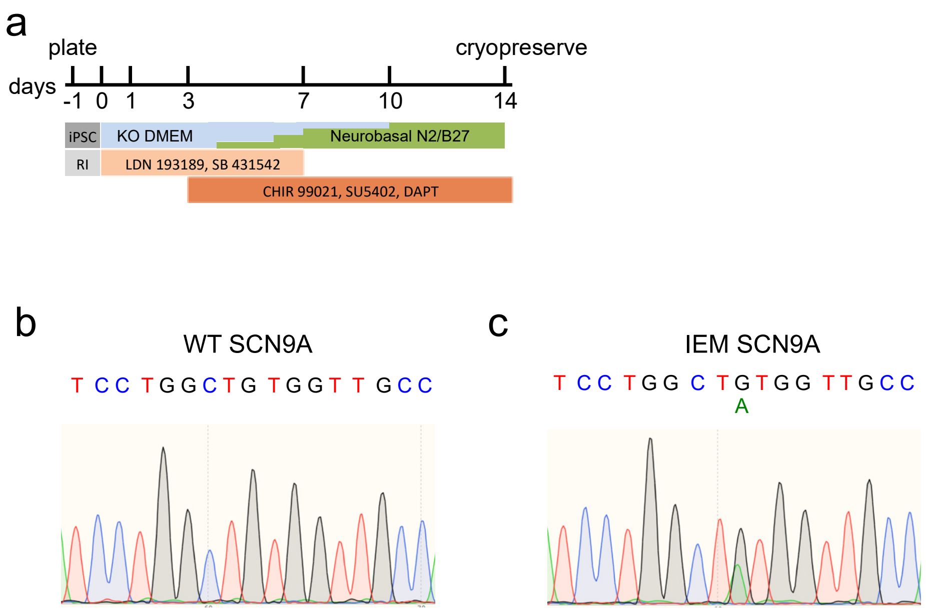


*Supplementary Figure 1*: *Generation of hiPSCdSN and validation of SCN9A mutation in patient hiPSC* **a)** Differentiation paradigm for the generation of human hiPSC-derived sensory neurons. hiPSC: induced pluripotent stem cells, RI: ROCK-Inhibitor **b, c)** Genotyping of wild type (b) and IEM-patient (c) hiPSC for V400M mutation in *SCN9A* gene. WT: wild type, IEM: inherited erythromelalgia.

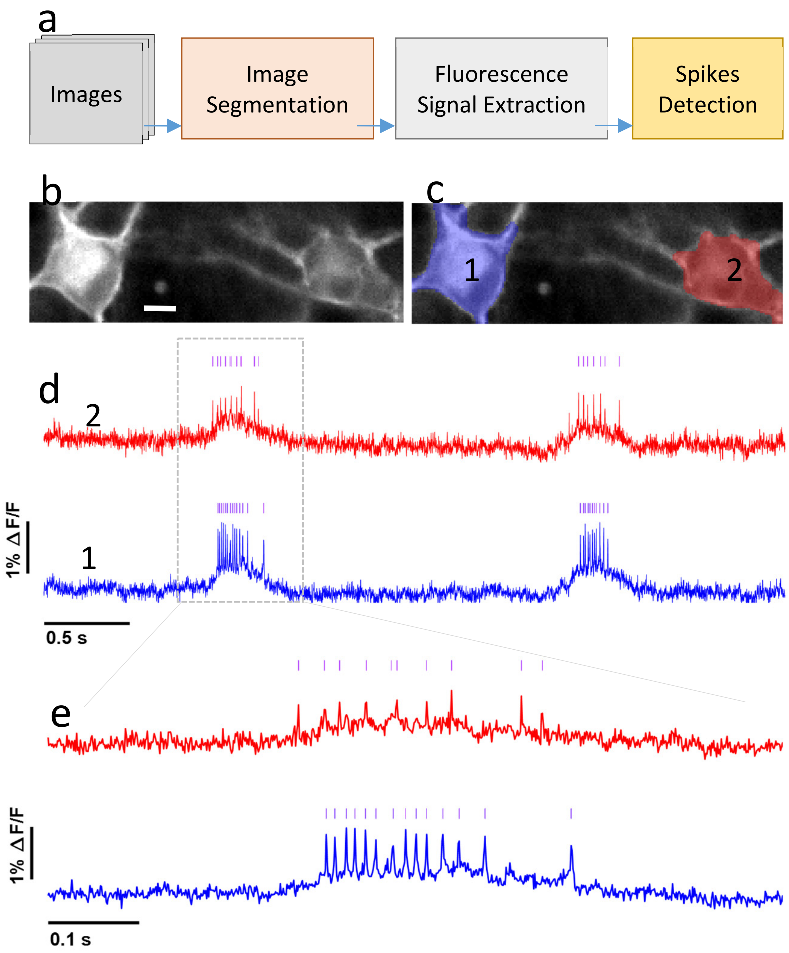


*Supplementary Figure 2*: *Cell detection pipeline*
The video frames were processed using a custom-written app in Matlab (MathWorks, USA). (a) The stack of frames was loaded as grayscale images. An image file from the whole stack was selected as a reference frame for further image processing and finding the ROIs of the cells. The reference frame was filtered using a 2D Gaussian kernel with a standard deviation of 1 for removing noise. The grayscale image was binarized using the adaptive threshold method. (b,c) The cells in the field of view were segmented after applying morphological image processing operations and then the reference ROIs were automatically generated. (d,e) The mean intensity in the ROI of each frame in the whole stack was calculated and used as the fluorescence signal of the corresponding cell. The extracted intensity for each frame was plotted to obtain the fluorescence signal in the time domain. A linear fit was applied to the fluorescence trace for baseline correction. Then, the intensity values were converted into ΔF/F (%) scale. Spikes were detected using a threshold value of 3-5 standard deviation above the mean.

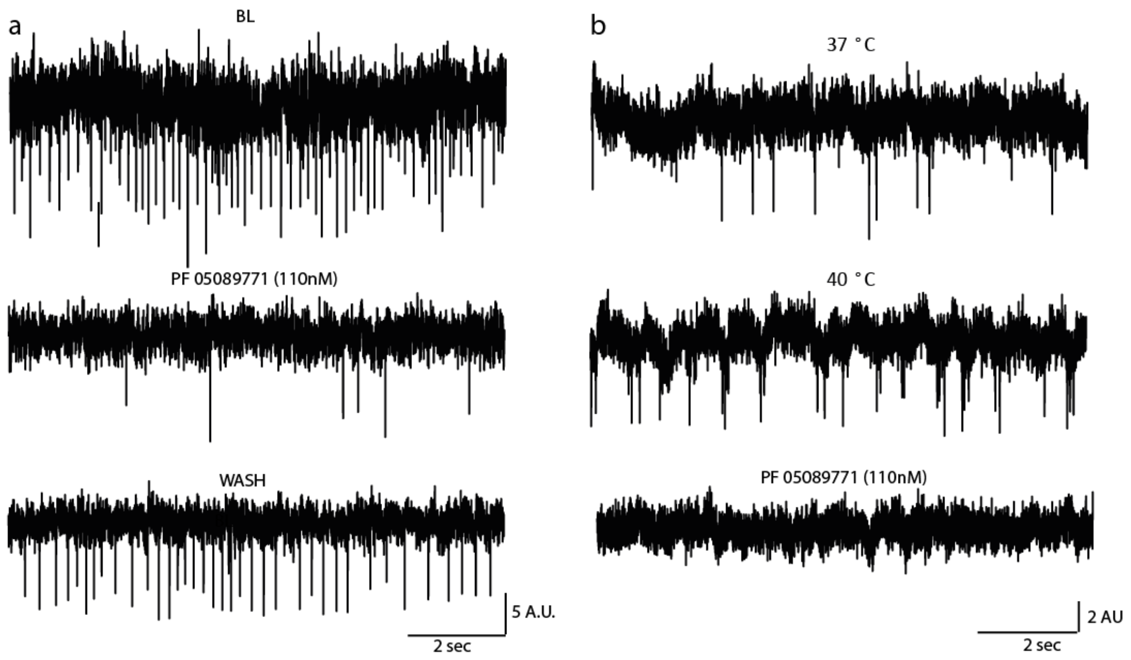


*Supplementary Figure 3*: Nav1.7 blocker reverses the hyperactive phenotype in EM patient hiPSCdSNs. (a) Representative traces of AP firing at baseline (*upper traces*), after 10 minutes of application of the Nav1.7 inhibitor PF-05089771 (*middle traces*) and 10 minutes after wash. (b) Representative traces show sporadic AP firing at 37°C (*upper traces*) increased firing at 40°C (*middle traces*) and elevated sensitivity to Nav1.7 inhibition at elevated temperatures (*lower trace*).
